# Loneliness Among the Silent Generation and Baby Boomers: A Replicated Cross-Sectional Analysis

**DOI:** 10.1093/geroni/igaf122.3113

**Published:** 2025-12-31

**Authors:** Amin Hashemzehi, Peter Martin

**Affiliations:** Iowa State University, Ames, Iowa, United States; Iowa State University, Ames, Iowa, United States

## Abstract

A significant portion of older Americans feels lonely. The relationship between age and loneliness is unclear, and few studies have considered cohort effects. This study investigated whether belonging to the Silent Generation or to Baby Boomers predicts loneliness levels. Analyses were computed on data from waves 9 (2008, n = 5201) and 15 (2020, n = 3278) of the Health and Retirement Study (HRS). Loneliness was measured using the 11-item UCLA loneliness scale. At wave 9, a t-test indicated significantly higher loneliness in Baby Boomers (t = -5.99, p < .001). Belonging to the Silent Generation, being female, higher education, African American ethnicity, better subjective health, fewer ADL difficulties, frequent contact with children, friends, and cohabitating with a partner significantly predicted lower loneliness in the final blocked regression model testing four models. The final model explained 17.4% of the overall variance. At wave 15, the loneliness mean was not significantly higher in Baby Boomers (t = -1.58, p = .12). Nevertheless, belonging to the Silent Generation, fewer difficulties in ADL, frequent contact with children, friends, and cohabitation significantly predicted lower levels of loneliness in the final regression model, explaining 15.4% of the variance. Cohort effects persisted, with Baby Boomers experiencing higher loneliness levels, highlighting the importance of cohort-specific factors. Future studies should explore period effects like social media and COVID-19, and the possible confounding role of age in cohort differences. Developing community support programs for Baby Boomers that emphasize social engagement and raising awareness about intergenerational interactions may be beneficial.

